# Posttraumatic Stress and Traumatic Brain Injury: Cognition, Behavior, and Neuroimaging Markers in Vietnam Veterans

**DOI:** 10.3233/JAD-221304

**Published:** 2023-10-10

**Authors:** Sofia Marcolini, Philine Rojczyk, Johanna Seitz-Holland, Inga K. Koerte, Michael L. Alosco, Sylvain Bouix, for the Department of Defense Alzheimer’s Disease Neuroimaging Initiative

**Affiliations:** a Department of Neurology and Alzheimer Center, University Medical Center Groningen, Groningen, The Netherlands; b Department of Psychiatry, Psychiatry Neuroimaging Laboratory, Brigham and Women’s Hospital, Harvard Medical School, Boston, MA, USA; c cBRAIN, Department of Child and Adolescent Psychiatry, Psychosomatics, and Psychotherapy, University Hospital, Ludwig Maximilian University Munich, Germany; d Department of Neurology, Boston University Alzheimer’s Disease Research Center, Boston University CTE Center, Boston University Chobanian & Avedisian School of Medicine, Boston, MA, USA; e Department of Software Engineering and Information Technology, École de Technologie Supe´rieure, Montre´al, Canada

**Keywords:** Alzheimer’s disease, Alzheimer’s Disease Neuroimaging Initiative, amyloid-β, diffusion magnetic resonance imaging, follow-up studies, risk factors

## Abstract

**Background:**

Posttraumatic stress disorder (PTSD) and traumatic brain injury (TBI) are common in Veterans and linked to behavioral disturbances, increased risk of cognitive decline, and Alzheimer’s disease.

**Objective:**

We studied the synergistic effects of PTSD and TBI on behavioral, cognitive, and neuroimaging measures in Vietnam war Veterans.

**Methods:**

Data were acquired at baseline and after about one-year from male Veterans categorized into: PTSD, TBI, PTSD+TBI, and Veteran controls without PTSD or TBI. We applied manual tractography to examine white matter microstructure of three fiber tracts: uncinate fasciculus (*N* = 91), cingulum (*N* = 87), and inferior longitudinal fasciculus (*N* = 95). ANCOVAs were used to compare Veterans’ baseline behavioral and cognitive functioning (*N* = 285), white matter microstructure, amyloid-β (*N* = 230), and tau PET (*N* = 120). Additional ANCOVAs examined scores’ differences from baseline to follow-up.

**Results:**

Veterans with PTSD and PTSD+TBI, but not Veterans with TBI only, exhibited poorer behavioral and cognitive functioning at baseline than controls. The groups did not differ in baseline white matter, amyloid-β, or tau, nor in behavioral and cognitive functioning, and tau accumulation change. Progression of white matter abnormalities of the uncinate fasciculus in Veterans with PTSD compared to controls was observed; analyses in TBI and PTSD+TBI were not run due to insufficient sample size.

**Conclusions:**

PTSD and PTSD+TBI negatively affect behavioral and cognitive functioning, while TBI does not contribute independently. Whether progressive decline in uncinate fasciculus microstructure in Veterans with PTSD might account for cognitive decline should be further studied. Findings did not support an association between PTSD, TBI, and Alzheimer’s disease pathology based on amyloid and tau PET.

## INTRODUCTION

Posttraumatic stress disorder (PTSD) and traumatic brain injury (TBI) are highly prevalent among military Veterans [1, 2] and are associated with short- and long-term behavioral disturbances and cognitive decline [3–5]. Interestingly, many long-term symptoms related to PTSD and TBI can also be observed in individuals with Alzheimer’s disease (AD). Overlapping symptoms include cognitive decline in attention, memory, and language performance [6, 7], neuropsychiatric symptoms such as depression, apathy, or agitation [8, 9], and impairment in activities of daily living [6].

Researchers have speculated that common underlying neuropathologies can explain the symptomatic overlap between PTSD, TBI, and AD [10–14]. Specifically, the accumulation of amyloid-β (Aβ) plaques and tau neurofibrillary tangles have been suggested as two of the mechanisms underlying PTSD, TBI, and AD [15, 16]. Neuritic Aβ plaques and tau deposits are indeed hallmarks of AD pathology [17, 18]. Animal research showed that psychological and physiological stress leads to Aβ genesis, proposing a potential link between Aβ plaques, PTSD, and TBI [19–22]. PTSD studies in humans, however, are limited and do not support the idea of an accumulation of Aβ plaques or tau in PTSD [23–26]. *In vivo* studies in TBI presented mixed results, with some amyloid and tau positron emission tomography (amyloid-PET and tau-PET) studies reporting increased Aβ and tau after TBI [27, 28]. Nevertheless, others did not find Aβ plaques and tau accumulation in individuals who experienced a TBI, implying that the effect might depend on individual risk factors such as genetic or medical conditions and the type of exposure and timing of TBI [25, 29]. A recent study also found no association between remote mild TBI and cortical Aβ burden [30].

Recent studies also suggested that common white matter pathologies might underly AD, PTSD, and TBI. Diffusion magnetic resonance imaging (dMRI) studies showed that white matter microstructural abnormalities are present in early stages of AD, therefore models using dMRI may provide biomarkers of white matter neurodegeneration in the earliest stages of AD progression [31–33]. Notably, the same fiber tracts related to AD development are also impaired in individuals with PTSD or TBI [34–43]. While findings are somehow inconsistent [44, 45], the uncinate fasciculus (UF), the cingulum (CI), and the inferior longitudinal fasciculus (ILF) have all been associated with cognitive decline and aging in PTSD, TBI, and AD [34–36, 38, 40, 41, 46–49]. Additionally, the UF and CI were found to be the most important tracts in distinguishing groups with PTSD and trauma-exposed controls [50]. The UF connects the anterior temporal lobe with the lateral orbitofrontal cortex and is involved in emotion and memory regulation [51, 52]. The CI stretches from the orbital frontal cortex, along the corpus callosum, to the temporal lobe and pole and is involved in executive control, episodic memory, and emotion [53]. The ILF connects the occipital and temporal lobes and is critical for visual memory, perception, reading, and language functions [51].

While PTSD, TBI, and AD share some neuropathological and clinical features, it remains elusive whether PTSD [13] and TBI [25, 29, 54] predispose to AD development. A recent meta-analysis did not find a relationship between PTSD diagnosis and subsequent dementia development [55]. Similarly, prospective studies failed to detect associations between TBI and autopsy-confirmed AD [29, 54]. On the other hand, there is evidence showing that Veterans who experience psychological and physical trauma are at increased risk of developing AD [56, 57]. These findings demonstrate that we are far from understanding the link between PTSD, TBI, and vulnerability for AD, and further research is needed considering the increasing number of aging individuals in general and aging Veterans in particular.

The Department of Defense (DOD) funded a project involving Vietnam war Veterans as part of the Alzheimer’s Disease Neuroimaging Initiative (ADNI) to meet this critical need (http://adni.loni.usc.edu). AD markers were measured in Veterans decades after trauma exposure twice: at baseline and after a one-year follow-up period [58]. A previous cross-sectional ADNI-DOD study revealed lower cognitive functioning in Veterans with PTSD, TBI, and PTSD+TBI compared to Veteran controls; however, impairments in cognitive functioning were not related to Aβ deposition [25]. In that study, when using a 1.11 cut-off for amyloid positivity, the PTSD group had lower odds of amyloid positivity compared to controls, while the TBI group did not [25]. Another recent study from the ADNI-DOD group found no evidence of increased Aβ, tau, or neurodegeneration biomarkers in either TBI or PTSD [59]. Nevertheless, other ADNI-DOD studies reported a negative effect of PTSD and TBI on white matter microstructure throughout the brain and tau pathologies [60, 61].

The present study builds on previous work [25, 59] to achieve two primary goals. First, we compare baseline measures of behavioral functioning, cognition, and neuroimaging markers that have previously been related to AD between Veterans with PTSD, TBI, PTSD+TBI, and Veteran controls. Next, we examine longitudinal changes in measurements over the one-year follow-up period to test for signs of progressive aging in Veterans with PTSD, TBI, PTSD+TBI, and Veteran controls. The current study extends previous ADNI-DOD studies by analyzing dMRI data using a methodology which allows a more sensitive reconstruction of white matter tracts as well as reporting on different neurobehavioral measures.

## METHODS

### Study design and the ADNI

The present study used cross-sectional data and for a subset longitudinal data obtained from a multicenter trial of the ADNI database, launched by the DOD in 2012 to study dementia risk factors in Veterans. Data were downloaded in April 2020. All data available in this study have been previously collected by the ADNI-DOD group. Our contribution has been in analyzing the diffusion-weighted images using a new methodology as described in the Diffusion MRI Image Processing section.

Vietnam War Veterans of age 60–80 years old were identified and contacted through Veterans Affairs records. They underwent an initial telephone screening, if eligible a clinical interview, and if still eligible they underwent baseline and follow-up visits at one of the DOD clinic sites. Study procedures, including the recruitment process, have already been published elsewhere (ADNI-DOD Procedures Manual, http://www.adni-info.org). Assessments for cognitive, behavioral, dMRI, and amyloid- and tau-PET data have been conducted at baseline and for cognitive, behavioral, dMRI, and tau-PET after a one-year follow-up. The follow-up period varied among individuals and type of assessment. To reduce this variability we removed extreme cases (corresponding to the 3rd quartile + 3* interquartile range and 1st quartile – 3* interquartile range), resulting in the inclusion of participants with a time interval between 7.5 months and 1.6 years. The distribution of time intervals for each measure is reported in the Results section.

### Participants

Three hundred and fifteen male Vietnam war Veterans between 60–80 years of age were included in the ADNI-DOD study. According to the ADNI-DOD protocol, participants were excluded if they reported a history of psychosis, bipolar disorder, a history of alcohol or substance abuse within the past five years (Diagnostic and Statistical Manual, Version IV, Text Revision, DSM IV-TR criteria); neurological disorders in the past five years and unstable (<four months) somatic conditions (e.g., cardiovascular diseases, hepatic, renal, pulmonary, metabolic diseases, or cancer). Moreover, participants were excluded when contraindications for PET (i.e., history of severe drug allergy or hypersensitivity) or MRI (i.e., aneurysm clips, metal implants, claustrophobia) applied.

Group-specific inclusion and exclusion criteria are described below:

1) PTSD participants had to meet the criteria for current or lifetime PTSD according to the Structured Clinical Interview 1 (SCID 1) of the DSM IV-TR, a minimum current Clinician Administered PTSD Scale (CAPS) score of 50, and symptoms related to the Vietnam war trauma, which were identified and verified via medical records and telephone assessments. Participants of this group were excluded if they had a documented history of TBI defined as in group 2.
2) TBI participants were required to have a documented history of non-penetrating TBI acquired during the military period in Vietnam. TBI history was identified from the DOD or Veterans Affairs records. Considering that the Glasgow Coma Scale was not available during the Vietnam war, the study coordinators used an operational definition of TBI based on diagnostic codes potentially related to TBI and available in medical records of the time, such as brain hemorrhage and traumatic brain disease (the specific TBI-related diagnostic codes can be found in Supplementary Table 1 of Weiner et al. (2023) [59]). Their operational definition also entailed non-penetrating head injury with amnesia, and/or loss of consciousness for 5 min, and/or alteration of mental state for > 24 h (also see ADNI-DOD protocol [62]). Participants of this group were excluded if they met PTSD diagnostic criteria (current or lifetime) defined as for group 1.
3) Participants of the PTSD+TBI group had to have both PTSD (diagnosed with SCID or CAPS) and TBI (identified from DOD or Veterans Affairs records) following the criteria for PTSD and TBI described in groups 1 and 2.
4) Veteran control participants were required to have neither a history of PTSD nor of TBI.

**Fig.1:**
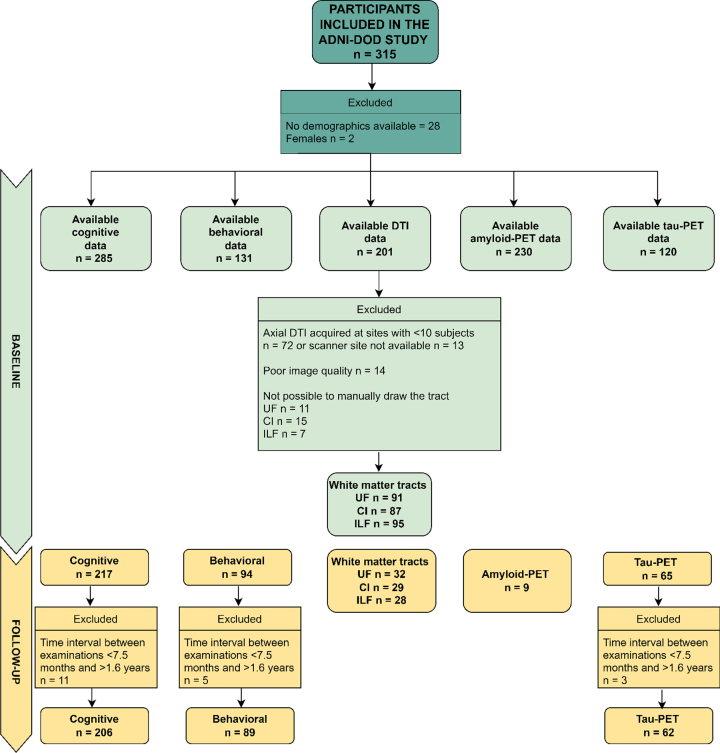
Flow Diagram of Available Data Used in the Current Study (Cognitive and Behavioral Data, Axial DTI, Amyloid-PET, Tau-PET at Baseline and Follow-up). Axial DTI, Axial Diffusion Tensor Imaging (as named in the ADNI data repository); CI, cingulum ILF, inferior longitudinal fasciculus; UF, uncinate fasciculus; PET, positron emission tomography. The limited sample of Amyloid-PET data at follow-up is due to budget restrictions that did not allow the ADNI-DOD team to repeat the florbetapir examinations as reported in their protocol (https://adni.loni.usc.edu/wp-content/uploads/2013/09/DOD-ADNI-IRB-Approved-Final-protocol-08072012.pdf, page 39).

We excluded females (only 2 participants) and everyone with no demographic data for the present study. In addition, dMRI data were excluded if acquired at sites where less than ten participants had been scanned, leading to a smaller sample than implied in the recruitment protocol. This approach allowed us to more robustly correct for site differences in the statistical analyses, since the usage of different scanners and protocols has been previously shown to introduce significant variability in results [63, 64]. Considering that the sample varies for each analysis, we refer the reader to Fig. 1 which shows the available data utilized in each analysis run in the present study. Participants were classified by the ADNI-DOD team (file “VAELG.csv”) into four groups: 1) Veterans with PTSD, 2) Veterans with TBI, 3) Veterans with PTSD+TBI, and 4) Veterans without a history of TBI or PTSD (Veteran controls).

### Demographics and clinical measures

Sample characteristics, including age, education, lifetime CAPS score, genotype, ethnicity, race, primary language, current work status, handedness, marital status, and psychiatric medication use, were assessed.

### Cognitive and behavioral assessment

Trained psychometricians administered cognitive tests and behavioral assessment tools following standardized procedures. Cognitive tests assessed the domains of confrontation naming (Boston Naming Test, BNT [65]), executive functioning (Trails Making Test B-A, TMT B-A [66]), and episodic memory (30-minutes delay Auditory Verbal Learning Test, AVDEL30 [67]). The behavioral assessment consisted of assessing neuropsychiatric disturbances (delusions, hallucinations, agitation, dysphoria, anxiety, apathy, irritability, euphoria, disinhibition, aberrant motor behavior, night-time behavior disturbances, appetite and eating abnormalities) measured using the Neuropsychiatric Inventory (NPI) total score (calculated adding up all domain scores which were obtained multiplying frequency by severity) [68] and functioning in activities of daily living evaluated with the Functional Assessment Questionnaire (FAQ) [69]. Tests’ raw scores were used. Higher scores on the BNT and AVDEL30 and lower scores on the TMT B-A indicate better cognitive performance. Higher scores on the NPI and FAQ indicate worse behavioral functioning.

### PET and MRI neuroimaging

#### PET acquisition

Amyloid-PET images were acquired using the [^18^F]-AV45 radiotracer (florbetapir) and tau-PET using [^18^F]-AV1451 (flortaucipir). Injection (dosage of 370 MBq, 10 mCi±10% bolus) was followed by a resting-uptake phase of 50 min for [^18^F]-AV45 and 75 min for [^18^F]-AV1451. Participants then entered the tomograph, and four emission frames of 5 min each were acquired for amyloid-PET. For tau-PET, six frames of 5-min duration were performed. Amyloid-PET images were 3D reconstructed using Iterative (fully 3D Iteration; four iterations; 20 subsets) with a grid of 128×128, FOV: 256×256 mm, slice thickness: 3.27 mm [70]. This study used the amyloid and tau PET data previously analyzed by the Jagust laboratory at the University of California Berkeley. For amyloid-PET we used the file “UCBERKELEYAV45_20190808” available on the ADNI database. For all statistical analyses the following areas were selected to measure brain Aβ: frontal (weighted florbetapir mean in frontal regions, “FRONTAL_SUVR”), cingulate (weighted florbetapir mean in anterior/posterior cingulate regions, “CINGULATE_SUVR”), parietal (Weighted florbetapir mean in lateral parietal regions, “PARIETAL_SUVR”), temporal (Weighted florbetapir mean in lateral temporal regions, “TEMPORAL_SUVR”), and composite florbetapir cortical standardized uptake value ratio, SUVR (“COMPOSITE_SUVR, Summary florbetapir cortical SUVR. Weighted florbetapir mean in frontal, cingulate, parietal, and temporal regions, defined by Freesurfer”). For tau-PET, we used a summary measure of the temporal region from the file “UCBERKELEYAV1451_MRIFREE_20210506” (temporal flortaucipir standardized uptake value ratio, “METATEMPORAL_SUVR”, weighted AV1451 mean of temporal summary region, with regions defined by neuromorphometrics and listed elsewhere [71]). The PET data available on the ADNI database is already intensity normalized; although Jagust’s laboratory recommends to normalize again the SUVRs with a reference region, as explained in details elsewhere [73]. In Supplementary Table 3, we provide results also for composite Aβ and tau SUVR relative to a reference region.

#### MRI acquisition

MRI data were obtained using 3 Tesla scanners with standardized procedures across different ADNI sites (*N* = 24, this number corresponds to the institutions where the MRI acquisition took place and not to the clinical ADNI site). The current study used axial diffusion tensor imaging (“Axial DTI”) scans. Data was obtained using axial spin-echo sequence (TR/TE=9050 ms/minimum, matrix = 256×256×59, resolution = 1.37×1.37×2.7 mm35, and b-value=5×b0 + 41 directions with *b* = 1000 s/mm^2^). Further information on MRI acquisition is available in the DOD ADNI procedures manual (Version 3.0 Augustus 28, 2017) [72].

### Diffusion MRI image processing

#### Pre-processing

Pre-processing steps were performed using an in-house pre-processing pipeline [73], correcting for head motion, eddy current distortion, and generating brain masks. Image quality was visually checked for artifacts using 3D Slicer [74], leading to the exclusion of fourteen participants (five controls, three PTSD+TBI, three TBI, and three PTSD). Brain masks were visually inspected and manually corrected where necessary using 3D Slicer.

#### Whole brain tractography

We reconstructed fiber pathways utilizing whole-brain unscented Kalman Filter (UKF) tractography [75]. This method reconstructs multiple fiber orientations within a voxel and is robust to noise [76, 77]. Moreover, UKF tractography can support multiple dMRI models, including single and multi-tensor models, free water (FW) imaging, or neurite orientation dispersion and density imaging (NODDI). For this study, we used a 2-tensor plus free water model (2 tensor + free water model, 1 seed per voxel, minimum seed fractional anisotropy (FA)=0.18, terminating FA = 0.15, terminating mean signal = 0.10), which has been shown to provide accurate fiber tracking [77, 78]. This approach was chosen since UKF tractography has been shown to be one of the most accurate tractography techniques especially compared to single-tensor methods [77, 79, 80]. In short, the orientation of a local fiber is traced using the estimation at the previous position to direct the estimation at the current position. Kalman filter provides a recursive estimation which greatly improves the accuracy of fiber orientations [74].

#### Fiber bundle extraction

The UF, CI, and ILF of both hemispheres were selected as tracts of interest. A description of the three tracts and their function is reported in Table 1. The tracts were manually extracted following procedures outlined elsewhere [81, 82]. Briefly, individual fibers from full-brain tractograms were identified as part of a tract of interest if they traverse manually defined inclusionary regions but do not traverse exclusionary regions. The rationale for choosing manual tracts of interest estimation was that automated tract identification is often anatomically inaccurate in populations with significant damage to connections [83]. While manual approaches can be impractical with large cohorts and rely on human expertise, manual fiber bundle identification results in low values of true negatives [82] and has commonly been used with patient populations [84–86].

**Fig. 2:**
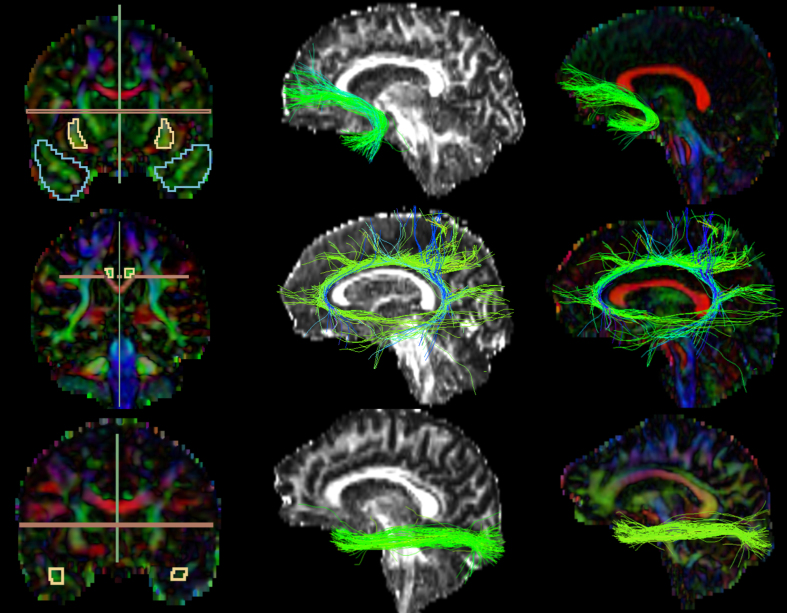
Extracted Tracts. The first images on the left column partially represent the manual drawings of the inclusion and exclusion areas; 1^st^ row) Uncinate Fasciculus: blue (temporal area) and yellow (external capsule) inclusion, green and red exclusion; 2^nd^ row) Cingulate: yellow inclusion, red and green exclusion; 3^rd^ row) Inferior Longitudinal Fasciculus: yellow inclusion (temporal area; occipital not shown in the picture), green and red exclusion. The images on the second and third columns represent the extracted tracts shown over the FA and color-by-orientation maps, respectively.

FA and color-by-orientation maps were calculated from the pre-processed dMRI data and used for the manual delineation of inclusionary and exclusionary areas according to Catani & de Schotten’s (2008) rules [81]. Specifically, for the UF, two inclusion areas (external capsule and temporal areas) and three exclusion areas (in the mid-sagittal, mid-coronal, and mid-axial slice) were drawn (Fig. 2, first row). For the CI, one inclusion region of interest (ROI) and two exclusion areas (in the axial and sagittal slice) (Fig. 2, second row). For the ILF, two inclusion ROIs (occipital and temporal areas) and two exclusion areas (in the mid-sagittal and axial slice) were drawn (Fig. 2, third row). Following the manual drawings, fiber bundles were extracted using the “Tractography ROI Selection” function in 3D Slicer with the whole-brain tractography as input and the manual drawings as label map. To establish the reliability of the procedure, three raters independently performed manual drawing delineation on four study cases.

#### dMRI measure extraction

The UKF tractography algorithm estimates parameters describing the 2 tensor+free water model for each point along each fiber tract. FW-corrected FA is a more accurate measure compared to traditional FA, especially when modeling tissue structure in aging populations [87], where atrophy and thus partial volume effects are more prevalent [78, 88–90].

We extracted the mean FA values of the primary tensor of each fiber bundle using the “Tractography Measurements” module of 3D Slicer and averaged left and right hemisphere measures. FA is the most widely used diffusion measure and is considered sensitive to microstructural changes, such as myelinization and axonal density.

**Table 1:** Extracted Tracts and Relative Function

Tract	Location and function
Uncinate Fasciculus	The UF is a ventral associative bundle that connects the anterior temporal lobe with the lateral orbitofrontal cortex [146, 147]. It is considered (i) part of the limbic system [146], (ii) involved in emotion and memory [51], and (iii) object and face naming [52].
Cingulate	The CI is a medial associative bundle that runs from the orbital frontal cortex, along the corpus callosum, to the temporal lobe and pole [146]. It is part of the limbic system and involved in executive control, episodic memory, emotion, and pain [53].
Inferior Longitudinal Fasciculus	The ILF is a longitudinal bundle connecting the occipital and temporal lobes [146]. It is involved in face recognition [148], visual perception [149], reading [150], visual memory [151], and other language functions [51].

#### Fiber bundle extraction reliability

We measured the reliability of the manual tracts of interest definition procedure by having the primary rater and two additional raters perform tract of interest tracing in four study cases. Inter-rater reliability for the three tracts was calculated using a two-ways interclass correlation (ICC) with absolute agreement. The guidelines by Koo & Li (2016) were used to define the agreement, with ICC < 0.50 meaning poor agreement, 0.50–0.75 moderate, 0.75–0.90 good, >0.90 excellent. Inter-rater reliability resulted to be good or excellent for FA (UF: ICC = 0.80, CI: ICC = 0.82, ILF: ICC = 0.95).

### Statistical analysis

Statistical analyses were performed using the Statistical Package for Social Sciences (SPSS) Version 27 [91]. Means, standard deviations, and one-way analyses of variance (ANOVA) were computed for continuous variables; percentages and chi-square tests for categorical variables.

#### Group comparisons: cognitive, behavioral, and neuroimaging measures

To test for group differences (PTSD versus TBI versus PTSD+TBI versus Veteran controls) in cognitive functioning (BNT, TMT B-A, AVDEL30) and neuroimaging measures (white matter: UF, CI, and ILF FA; amyloid and tau PET: composite florbetapir cortical SUVR and flortaucipir temporal SUVR) twelve analyses of covariance (ANCOVAs) were conducted. For differences in behavioral functioning (NPI, FAQ), two Kruskall-Wallis tests were conducted to account for the skewness of the data (skewness was respectively 3.20 and 2.11); for all other analyses skewness lied between –2 and+2 [92] Lower scores on the BNT and AVDEL30, higher scores on the TMT B-A, NPI, and FAQ, decreased white matter FA, and higher composite florbetapir cortical SUVR and flortaucipir temporal SUVR were expected in Veterans with PTSD and/or TBI groups compared to the Veteran controls.

#### Group comparisons: cognitive, behavioral, and neuroimaging differences (follow-up -bBaseline)

To test for group comparisons in score differences between baseline and follow-up in cognitive functioning (BNT, TMT B-A, AVDEL30), behavioral functioning (NPI, FAQ), and UF, CI, and ILF white matter FA, eight ANCOVA models were implemented. White matter measures were compared only between Veterans with PTSD and Veteran controls due to the small sample size in the TBI and PTSD+TBI groups. Moreover, we did not compare composite florbetapir cortical SUVR difference scores between groups since follow-up measures were only available for nine participants due to budget restrictions as noted in Fig. 1. Scores’ raw differences were calculated (follow-up – baseline); scatterplots displaying the association between score differences and time interval are reported in Supplementary Figures 2 and 3. A negative score difference was expected in Veterans with PTSD and/or TBI for all dependent variables, except for a positive difference in TMT B-A, NPI, FAQ, and flortaucipir temporal SUVR, as compared to Veteran controls.

An overview of the statistical approach can be found in Supplementary Figure 1 and Supplementary Table 1. Note that analyses may have different sample sizes due to missing data. All ANCOVA models were corrected for age, education, and scanner site. Groups did not differ in other demographics (ethnicity, racial category, APOE *ɛ*4+) accordingly, these variables were not included as covariates. All models were corrected for multiple comparisons, setting a false discovery rate at 5% using the Benjamini-Hochberg method and considering a corrected *p* < 0.05 as significant.

## RESULTS

### Demographic and sample characteristics

A summary of the sample characteristics is displayed in Table 2. The one-way ANOVAs revealed statistically significant between-group differences in age (Veteran controls significantly older than PTSD), education (PTSD significantly lower education compared to Veterans with TBI and controls, and Veteran controls significantly higher than Veterans with PTSD+TBI), and CAPS score (PTSD having the highest score). Chi-square tests showed the lowest use of psychiatric medications among Veteran controls and the highest among Veterans with PTSD.

Of the sample with complete cognitive data at baseline, 236 participants were cognitively normal, 49 had a diagnosis of MCI (15 PTSD, 9 TBI, 23 PTSD+TBI, and 2 controls), and none had dementia. At month twelve, 178 were cognitively normal and 37 had MCI (11 PTSD, 9 TBI, 16 PTSD+TBI, and 1 control). Of the sample with available DTI data (considering the ILF sample), 90 were cognitively normal, 5 had a diagnosis of MCI (2 PTSD, 3 TBI, 1 PTSD+TBI, 0 controls), and none had dementia (this data was retrieved from the “DXSUM” spreadsheet).

### Group differences in cognitive and behavioral functioning

**Fig. 3:**
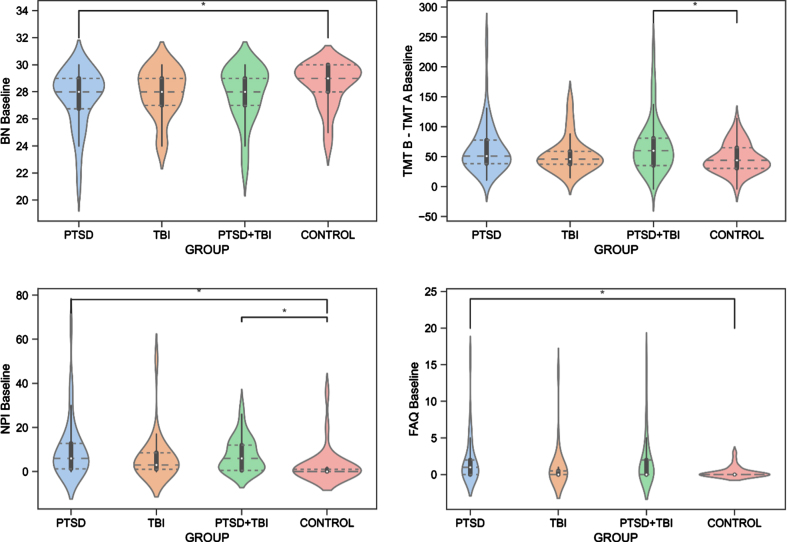
Group Differences in Cognitive and Behavioral Functioning. BN, Boston Naming Test, Naming Abilities; FAQ, Functional Assessment Questionnaire, Functioning in Activities of Daily Living; NPI, Neuropsychiatric Inventory, Neuropsychiatric Disturbances; PTSD, posttraumatic stress disorder; TBI, traumatic brain injury; TMT B – TMT A, Trail Making Test B-A, Executive Functioning.

The ANCOVAs revealed significant differences between the groups for the BNT test (F(3, 278) = 3.046, *p* = 0.043, *η*_p_^2^ = 0.040). Specifically, Veterans with PTSD had significantly worse (*p* = 0.020) confrontation naming abilities than Veteran controls. Moreover, the groups differed on the TMT B-A (F(3,275) = 3.125, *p* = 0.040, *η*_p_^2^ = 0.033), revealing lower performance of executive functioning in Veterans with PTSD+TBI compared to Veteran controls (*p* = 0.040). No group effect was seen for the AVDEL30 (*p* > 0.05), measuring delayed memory performance. The Kruskal-Wallis test showed group differences in the NPI (*H*(3) = 17.888, *[H]* = 0.12, *p* = 0.001) and FAQ (*H*(3) = 9.965, *[H]* = 0.06, *p* = 0.020). Specifically, Veterans with PTSD and PTSD+TBI showed more neuropsychiatric disturbances than Veteran controls (*p* < 0.001), and Veterans with PTSD displayed higher FAQ scores than Veteran controls (*p* = 0.030), reflecting worse functioning in activities of daily living. The results are reported in Table 3 and Fig. 3, and detailed sample sizes are reported in Table 3.

**Table 2:** Sample characteristics at baseline

		PTSD	TBI	TBI+PTSD	Control	Total	*p*
		*n* Mean±SD	*n* Mean±SD	*n* Mean±SD	*n* Mean±SD	*n* Mean±SD	*p*
Age (years) Education (years) Life CAPS		78 68.10±3.32 14.54±2.36 74.25±18.02	43 70.37±5.42 16.05±2.30 14.56±9.69	93 69.83±2.96 14.84±2.48 59.70±18.87	71 71.40±5.79 16.06±2.13 5.95±8.15	285 69.84±4.49 15.24±2.41 44.20±32.50	<0.001* <0.001* <0.001*
		%	%	%	%	%	*p*
Genotype	APOE *ɛ*4+	23.4%	32.5%	25.0%	27.1%	26.2%	0.745
Ethnicity	Hispanic	12.8%	4.7%	6.5%	5.6%	7.7%	0.476
	Caucasian	85.9%	93.0%	92.5%	94.4%	91.2%
	Unknown	1.3%	2.3%	1.1%	0%	1.1%
Race	American Indian	1.3%	0%	2.2%	1.4%	1.4%	0.550
	Asian	0%	0%	0%	4.2%	1.1%
	African American	7.7%	9.3%	8.6%	5.6%	7.7%
	White	84.6%	86.0%	81.7%	85.9%	84.2%
	More than one race	5.1%	4.7%	6.5%	1.4%	4.6%
	Unknown	1.3%	0%	1.1%	1.4%	1.1%
Language	English	97.4%	100%	97.8%	97.2%	97.9%	0.810
	Spanish	2.6%	0%	1.1%	1.4%	1.4%
	Other	0%	0%	1.1%	1.4%	0.7%
Work Status	Working	10.3%	18.6%	8.6%	19.7%	13.3%	0.113
	Retired	89.7%	81.4%	91.4%	80.3%	86.7%
Handedness	Right	88.5%	86.0%	91.4%	90.1%	89.5%	0.796
	Left	11.5%	14.0%	8.6%	9.9%	10.5%
Marital Status	Married	82.1%	86.0%	68.8%	85.9%	79.3%	0.168
	Widowed	3.8%	0%	8.6%	4.2%	4.9%
	Divorced	7.7%	11.6%	15.1%	5.6%	10.2%
	Never married	6.4%	2.3%	7.5%	4.2%	5.6%
Medication usage	No psychiatric med	43.5%	85.7%	69.1%	93.0%	71.5%	<0.001*
	Donezepil	0%	2.4%	0%	0%	0.4%
	Galantamine	1.4%	2.4%	0%	0%	0.8%
	Anti-depressants	44.9%	7.1%	28.4%	5.6%	23.2%
	Other psychiatric med	10.1%	2.4%	2.5%	1.4%	4.2%

CAPS, Clinician Administered PTSD Scale; GD, geriatric depression; med, medication; PTSD, posttraumatic stress disorder; SD, standard deviation; TBI, traumatic brain injury. *Significant differences in the one-way ANOVA (continuous variables) or Chi-square test (categorical variables).

**Table 3:** Cognitive, behavioral, and neuroimaging data at baseline

Analyses Dependent variables	PTSD			TBI	PTSD+TBI			Control		TOTAL	*p*	FDR corrected *p*	Post hoc PTSD- TBI	Post hoc PTSD- Control	Post hoc PTSD- PTSD+ TBI	Post hoc TBI- PTSD+ TBI	Post hoc TBI- Control	Post hoc Control- PTSD+TBI
ANCOVA	*n*	Mean±SD	*n*	Mean±SD	*n*	Mean±SD	*n*	Mean±SD	*n*	Mean±SD	*p*	*p* ^ +^	*p* ^ +^	*p* ^ +^	*p* ^ +^	*p* ^ +^	*p* ^ +^	*p* ^ +^
BNT	78	27.50±2.12	42	28.05±1.70	93	27.82±2.11	71	28.54±1.66	284	27.94±1.98	0.03*	0.04*	1.00	0.02*	1.00	1.00	0.99	0.27
TMT B-A	75	63.35±42.56	43	54.23±29.80	93	69.06±45.26	71	52.75±35.49	282	61.17±40.51	0.03*	0.04*	0.87	0.21	0.32	0.316	1.00	0.04*
AVDEL30	78	6.12±3.48	43	6.05±3.81	93	5.19±3.72	71	6.32±3.94	285	5.25±3.78	0.34	0.34
Kruskal Wallis	*n*	Mean Rank	*n*	Mean Rank	*n*	Mean Rank	*n*	Mean Rank	*n*	*p*	*p* ^ +^	*p* ^ +^	*p* ^ +^	*p* ^ +^	*p* ^ +^	*p* ^ +^	*p* ^ +^
NPI	41	77.43	22	64.66	43	73.15	25	38.46	131	<0.00*	0.00*	1.00	0.00*	1.00	1.00	0.10	0.00*
FAQ	41	74.74	22	57.32	43	71.02	25	50.66	131	0.01*	0.02*	0.30	0.03*	1.00	0.72	1.00	0.09
ANCOVA	*n*	Mean±SD	*n*	Mean±SD	*n*	Mean±SD	*n*	Mean±SD	*n*	Mean±SD	*p*
UF FA	31	0.58±0.34	8	.57±.05	23	.57±.04	29	.56±.04	91	.56±.04	.31
CI FA	30	0.51±0.48	8	.55±.05	23	.51±.04	26	.53±.04	87	.52±.05	.22
ILF FA	29	0.67±0.04	10	.68±.04	23	.66±.04	33	.66±.04	95	.66±.04	.36
Florbetapir frontal^1^	70	1.21±.16	32	1.23±.26	61	1.28±.25	67	1.28±.23	230	1.25±.22	.40
Florbetapir cingulate^1^	70	1.34±.18	32	1.35±.27	61	1.41±.25	67	1.41±.25	230	1.38±.23	.34
Florbetapir parietal^1^	70	1.24±.14	32	1.26±.30	61	1.30±.23	67	1.30±.25	230	1.28±.23	.60
Florbetapir temporal^1^	70	1.15±.14	32	1.18±.25	61	1.21±.21	67	1.22±.22	230	1.19±.20	.66
Florbetapir composite cortical^1^	70	1.24±.15	32	1.26±.26	61	1.30±.23	67	1.30±.23	230	1.27±.22	.57
Flortaucipir temporal^1^	38	1.15±.09	18	1.18±.08	37	1.14±.12	27	1.15±.08	120	1.15±.10	.68

AVDEL30, Auditory Verbal Learning Test 30 Minutes Delayed; BNT, Boston Naming Test; CI FA, cingulate fractional anisotropy; FAQ, Functional Assessment Questionnaire; FDR, false discovery rate; ILF FA_,_ inferior longitudinal fasciculus fractional anisotropy; NPI, Neuropsychiatric Inventory; PTSD, posttraumatic stress disorder; SD, standard deviation; SUVR, standardized uptake value ratio; TBI, traumatic brain injury; TMT B-A, Trail Making Test B-A; UF FA, uncinate fasciculus fractional anisotropy.*Significant differences in the Kruskal-Wallis tests; *p* + FDR corrected; ^1^SUVR. Data for BNT, TMT B-A, AVDEL30 is reported from models correcting for age and education. Sample for the TMT B-A and BNT differs since three subjects miss the TMT B score and one the BNT score. Data for the neuroimaging measures from models correcting for age, education, and scanner id.

### Group differences in neuroimaging measures

Differences in all sample characteristics among groups with and without available or analyzed DTI data were examined; groups did not differ in any demographic characteristics (*p* > 0.05), although participants without available or analyzed DTI data had higher lifetime CAPS, worse scores in the AVDEL30 test, had more PTSD+TBI and less control participants, and more participants with MCI diagnosis compared to participants with available or analyzed DTI data (results are reported in Supplementary Table 2). The ANCOVA analyses showed no significant differences in baseline FA for the three tracts (UF, CI, ILF) between Veterans with PTSD (*n* = 31), PTSD+TBI (*n* = 23), and Veteran controls (*n* = 29) [the group of Veterans with TBI only (*n* = 8) was not included due to the small sample size]. Moreover, no differences were shown in any of the florbetapir SUVR regions (frontal, cingulate, parietal, temporal, and composite cortical) nor in baseline flortaucipir temporal SUVR for all four groups (*p* > 0.05, Table 3).

### Group differences in cognitive, behavioral, and neuroimaging score differences (follow-up – baseline)

ANCOVAs were performed to test for between-group differences in the cognitive and neuroimaging score differences from baseline to follow-up. After the removal of extreme cases, the time interval between the two assessments was of 1.07 (0.16) years for the cognitive and behavioral assessments ranging from 0.79 to 1.6 years; of 1.13 (0.12) for the diffusion MRI assessments ranging from to 1.02 to 1.44 years, and of 1.13 (0.22) ranging from 0.75 to 1.72 for the tau PET assessment. No significant group differences for score changes of the BNT, TMT B-A, AVDEL30, NPI, FAQ, and flortaucipir temporal SUVR were shown (*p* > 0.05).

**Fig.4:**
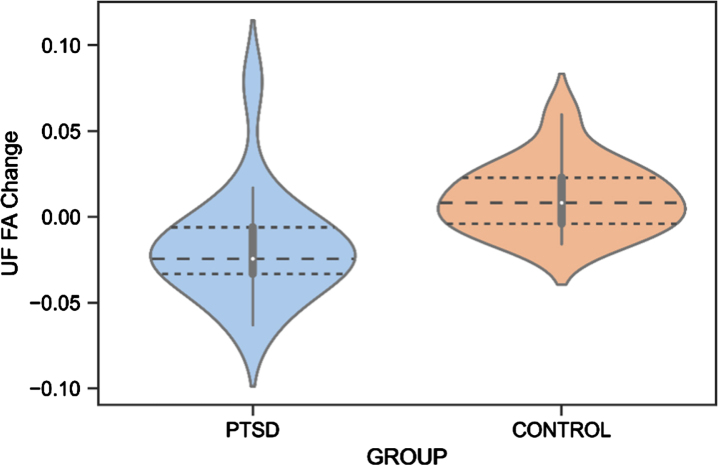
UF FA Change Difference Between Veterans with PTSD and Veteran Controls. PTSD, posttraumatic stress disorder; UF, uncinate fasciculus; FA, fractional anisotropy.

There was a significant difference between Veterans with PTSD compared to Veteran controls in score changes (follow-up – baseline) of the UF FA (*F*(1,27)=10.27, *p* = 0.001, $\eta_{p}^{2}$ = 0.28). Veterans with PTSD had a negative mean change of –0.018±0.031 (–2.79% difference) while Veteran controls had a positive mean change of .013±.020 (+2.05% difference) (Table 4, Fig. 4). No differences were revealed for FA of the ILF and CI (*p* > 0.05).

## DISCUSSION

The present study analyzed cognitive, behavioral, and neuroimaging data of a cohort of Vietnam war Veterans from the ADNI-DOD study. We showed that decades after trauma, 1) a diagnosis of PTSD or PTSD+TBI was associated with worse behavioral and cognitive functioning; 2) groups did not differ in white matter microstructure, Aβ and tau accumulation at baseline; 3) Veterans with PTSD showed a progressive decline in the UF microstructural integrity compared to Veteran controls over one year.

### Baseline differences in cognitive and behavioral functioning

Consistent with our hypothesis, Veterans with PTSD+TBI showed worse executive functioning and Veterans with PTSD showed worse confrontation naming abilities compared to Veteran controls. It has been suggested that Veterans with PTSD may have decreased cognitive reserve—the ability to cope with the harmful effects of pathology on cognitive functioning [93]. Poorer cognitive performance and lower cognitive reserve may then increase the risk for a manifestation of cognitive impairments. The pre-dementia stage is characterized by deficits in various cognitive domains but intact daily function [94], and a study indicated decreased language abilities among the most discriminant indicator of cognitive decline [95]. Lower general cognition was also found in a study using the ADNI-DOD sample in the PTSD and PTSD+TBI groups compared to Veteran controls [59]. In addition to deficits in cognitive functioning, our study also showed worse daily functioning among Veterans with PTSD and more neuropsychiatric disturbances in both PTSD and PTSD+TBI compared to Veteran controls. These findings align with previous studies [96, 97], and impairments in behavioral functioning have also been linked to an increased risk of AD development [98–101]. Therefore, cognitive and behavioral functioning should be closely monitored in Veterans with PTSD.

Contrary to our hypothesis, Veterans with TBI did not differ from Veterans with PTSD, PTSD+TBI, or Veteran controls in cognitive or behavioral functioning. Moreover, no differences were found between Veterans with PTSD or TBI and those with PTSD+TBI. It can be argued that PTSD and the pathological sequelae of TBI may play a role in the pathogenesis of cognitive decline rather than being a necessary or sufficient condition to develop dementia [13]. Furthermore, in the present study, Veterans belonging to the four cohorts were exposed to substantial distress and likely under-reported TBI events during deployment, making it difficult to discriminate between groups.

Some of the differences among groups might have been evened out due to all subjects having had combat exposure. Additionally, a previous meta-analysis has reported that significant heterogeneity was present among studies comparing samples of Veterans with and without PTSD [102]. Despite this limitation, comparing four groups of Veterans with different diagnoses allows one to specifically study patterns of cognitive reserve and different vulnerability among combats.

### No baseline differences in neuroimaging measures

In contrast to our hypothesis, we did not find significant differences in white matter measures, Aβ and tau at baseline between groups of Veterans with PTSD, TBI, and PTSD+TBI compared to Veteran controls. The null results for Aβ and tau suggest that the risk conferred by PTSD and TBI for cognitive decline might not be related to underlying AD pathology. While both PTSD [35, 36, 38, 39, 49, 103–116] and TBI [37, 44, 117–119] have repeatedly been linked to widespread abnormalities in white matter, some studies did not report significant differences between individuals with PTSD+TBI and controls [45, 120], consistent with our findings. Similarly, some studies reported differences in Aβ deposition [28, 70], while others, two of which also using ADNI-DOD data [25, 59], did not find any differences between Veterans with TBI [25, 29, 30, 121, 122] or PTSD [26] and Veteran controls. Our results on Aβ and tau are in accordance with a recent ADNI-DOD study that found no differences in Aβ and tau, as well as no differences in cerebrovascular disease measures [59]. Similarly, no differences were observed among individuals with a single moderate to severe TBI and controls in Aβ and tau burden years after the injury [123]. Analyzing these four groups separately allowed us to investigate whether PTSD or TBI as a loading factor to either condition led to a higher presence of AD biomarkers. This is particularly relevant since previous literature has shown that white matter abnormalities are exacerbated in PTSD+TBI [41, 124], also longitudinally [125]. It is possible that trauma-exposed Veteran controls present with an equally compromised brain structure as those who developed PTSD [126, 127]. Therefore, while our results are relevant for the Veteran population, future studies should investigate the impact of PTSD or TBI as loading factors in non-Veterans samples. Moreover, a TBI may lead to various presentations of brain abnormalities [6], which makes identifying a common pattern of imaging differences on a group level difficult. While it is beyond the scope of the current paper, considering mechanisms such as inflammation [128] or synaptic dysfunction [129] may be of value in future studies, given that AD onset comprises a cascade of different pathological mechanisms [130], not limited to Aβ deposition, tau accumulation, or white matter abnormalities.

**Table 4:** Cognitive, behavioral, and neuroimaging changes (Follow-up – Baseline)

Analysis Dependent variables	PTSD			TBI		PTSD+TBI		Control		TOTAL	*p*	FDR corrected *p*
ANCOVA Follow-up - baseline	N	Mean±SD	*n*	Mean±SD	*n*	Mean±SD	*n*	Mean±SD	*n*	Mean±SD	*p*	*p* ^ +^
BNT	58	0.05±1.76	36	0.39±1.57	57	0.26±1.51	55	0.16±1.23	206	0.20±1.52	0.56	0.56
TMT B-A	55	–0.67±33.83	36	–5.23±26.61	57	8.05±47.50	55	7.81±35.11	203	3.22±37.60	0.13	0.20
AVDEL30	58	–0.40±3.01	36	0.86±3.51	57	0.65±3.42	55	1.01±3.18	206	0.49±3.29	0.12	0.20
NPI	27	0.30±11.00	16	–0.50±6.00	25	–2.40±5.76	21	0.14±9.77	89	–0.46±8.61	0.58	0.58
FAQ	27	–0.26±2.10	16	–0.56±0.96	25	–0.96±2.37	21	0.48±1.21	89	–0.34±1.90	0.08	0.16
UF FA	17	–0.02±0.03	4	/	4	/	15	0.01±0.02	40	–0.00±0.03	0.00*	0.00*
CI FA	16	–0.01±0.03	4	/	4	/	13	–0.01±0.03	37	0.00±0.01	0.82	0.82
ILF FA	13	–0.00±0.04	4	/	4	/	15	0.01±0.04	36	0.01±0.04	0.47	0.71
Flortaucipir temporal^1^	19	–0.01±0.05	10	–0.03±0.03	20	0.01±0.04	13	0.01±0.03	62	–0.00±0.04	0.12	0.12

AVDEL30, Auditory Verbal Learning Test 30 Minutes Delayed; BNT, Boston Naming Test; CI FA, cingulate fractional anisotropy; FAQ, Functional Assessment Questionnaire; ILF FA, inferior longitudinal fasciculus fractional anisotropy; NPI, Neuropsychiatric Inventory; PTSD, posttraumatic stress disorder; SD, standard deviation; TBI, traumatic brain injury; TMT B-A, Trail Making Test B-A; UF FA, uncinate fasciculus fractional anisotropy.*Significant differences in the ANCOVA. *p* + FDR corrected. ^1^SUVR. Data for BNT, TMT B-A, AVDEL30, NPI, FAQ is reported from models correcting for age and education; data from FA corrected for age, education, and scanner id. Sample for the TMT B-A differs since three subjects miss TMT B at baseline. No differences were calculated for amyloid PET since insufficient follow-up data was available, due to budget restrictions as of protocol.

### White matter microstructure change in PTSD compared to controls

Monitoring longitudinal changes is critical when assessing the impact of PTSD and TBI on cognitive decline. We compared the differences in cognitive and behavioral functioning and white matter change from baseline to one-year follow-up between groups. While Veterans with PTSD and PTSD+TBI showed worsening in some of the cognitive and behavioral functioning domains, the groups did not differ significantly from each other, Veterans with TBI, or Veteran controls. Therefore, the findings suggest no accelerated decline of cognitive and behavioral functioning in Veterans diagnosed with PTSD, TBI, or PTSD+TBI for the studied age range and timespan.

Regarding neuroimaging measures, white matter changes have been suggested as a biomarker for the early identification of AD [131], and alterations in the UF, CI, and ILF white matter microstructure have previously been found in AD patients [131–133]. We reported that white matter microstructure of the UF decreased in Veterans with PTSD, while an improvement was found for the control group. Although an increase in white matter microstructure is not expected at an older age, Veteran controls may still be socially and cognitively active, potentially leading to neural restructuring. Due to the small sample size in these analyses, these preliminary findings should be interpreted cautiously. Previous studies reported abnormalities of the UF in individuals with PTSD [43, 113], although no study has yet investigated this longitudinally. Since the UF is involved in emotion processing, memory, and language functions [81], it is highly responsible for cognitive features commonly impacted in AD [134]. For example, a decrease in the number of fibers in the right UF has been associated with memory loss in individuals with mild cognitive impairments (MCI) [135]. Moreover, a relationship between decreased UF FA and poor performance on the Mini-Mental State Examination, a test for cognitive functioning in the elderly, was shown in individuals with probable AD [136]. Increased risk of dementia with considerably low FA of the UF was also found in individuals with amnestic MCI [137] and semantic dementia [138]. Our preliminary finding of a decrease in UF FA in Veterans with PTSD is thus in line with previous research and may indicate an early cognitive decline. Importantly, the UF finding might not be specific to AD but related to other neurodegenerative pathologies or to aging in general, considering that previous literature has found abnormalities in the UF also in frontotemporal dementia [139, 140].

Previous studies have found an association between cognitive reserve proxies and white matter integrity, supporting the involvement of white matter changes in processes of cognitive reserve. Cognitive reserve might also affect white matter microstructural changes over time, and eventually predispose veterans with PTSD to develop cognitive impairment [141, 142]. In a recent study, associations between cognitive reserve and white matter microstructure measured over time were found to differ by age in healthy older adults, suggesting that cognitive reserve has a neuroprotective role in middle age and shifts to a compensatory effect in older age [143].

No significant changes were observed from baseline to after one year in ILF and CI microstructure between Veterans with PTSD and Veteran controls. In a recent study involving individuals with MCI, the ILF was one of the tracts indicating the risk of conversion to AD [144]. In the current study, the ILF microstructure slightly decreased in the PTSD group and increased in the Veteran control group without reaching statistical significance, while a decrease in CI microstructure was seen in Veterans with PTSD and Veteran controls. Future research should investigate these trends using larger sample sizes.

### Limitations and future directions

We acknowledge several study limitations. Group differences in white matter microstructural changes of the TBI and PTSD+TBI groups were not investigated, given the small sample sizes. The same applied to follow-up amyloid-PET measures. In addition, we did not control for exposures to psychological or brain trauma or treatment during the follow-up year. Furthermore, additional information on the trauma (i.e., time since trauma, severity, number of TBIs) would have been beneficial for interpreting the findings. An additional limitation is that results might have varied depending on MCI diagnostic status. Although, we were unable to perform these subgroup analyses due to insufficient sample size within each PTSD and/or TBI group. However, removing MCI subjects would have led to biases in the selection of the sample, since individuals with TBI and PTSD might have a greater incidence of cognitive impairment. A major limitation of the present study is the small sample size for the white matter longitudinal analysis, calling for future studies validating these preliminary findings with a larger sample. Nevertheless, the current study provides meaningful insights into the longitudinal effects of PTSD and TBI on cognitive and behavioral functioning and brain structure that may indicate AD development.

Additional follow-up analyses are needed to validate the result of progressive white matter decline in Veterans with PTSD. This would allow conclusions on trajectories that cannot be reliably derived from only two assessment time points. Studies repeating assessments with a longer follow-up time are needed to assess whether progressive changes in cognition are seen after a longer period than one year. Although, considering the mean age of our sample, changes in AD markers can be expected after one year. Furthermore, accompanying Veterans with interventions to alleviate PTSD and TBI-related symptoms would be highly meaningful when studying the potential preventive effects of stress reduction and cognitive training for dementia onset. Moreover, a healthy control group of non-Veterans is needed to validate our findings. Lastly, future studies should consider assessing resilience predisposing factors (e.g., perceived health, sex, trait self-enhancement [145]) and investigate whether these influence the advent of cognitive decline in the Veteran population.

### Conclusion

PTSD and PTSD+TBI negatively impact behavioral and cognitive functioning in the aging Veteran population. Moreover, our findings suggest a decrease in UF white matter microstructure in Veterans with PTSD after one year. Differences in baseline neuroimaging measures and behavioral and cognitive change were not identified. Therefore, while PTSD and PTSD+TBI confer risk for cognitive decline, our results do not support a direct link with AD pathology given a lack of difference in episodic memory, Aβ, and tau. Instead, longitudinal white matter changes might account for the decline, but this preliminary result should be further investigated. We conclude that Veterans with PTSD and PTSD+TBI need to be monitored and treated adequately to prevent behavioral and cognitive decline.

## Supplementary Material

Supplementary Material

## Data Availability

Data used to prepare this article were obtained from the Alzheimer’s Disease Neuroimaging Initiative (ADNI) database (http://adni.loni.usc.edu).
