# Supplementary material for: Posttraumatic Stress and Traumatic Brain Injury: Cognition, Behavior, and Neuroimaging Markers in Vietnam Veterans
Source: J Alzheimers Dis. 2023 Oct 10;95(4):1427–48. doi: 10.3233/JAD-221304 (PMC10578246; doi:10.3233/JAD-221304)
Supplement: Supplementary Material [file jad-95-jad221304-s001.pdf]

Supplementary Material

Posttraumatic Stress and Traumatic Brain Injury: Cognition, Behavior, and Neuroimaging Markers in Vietnam Veterans

Supplementary Figure 1. Schematic Overview of the Statistical Approach.

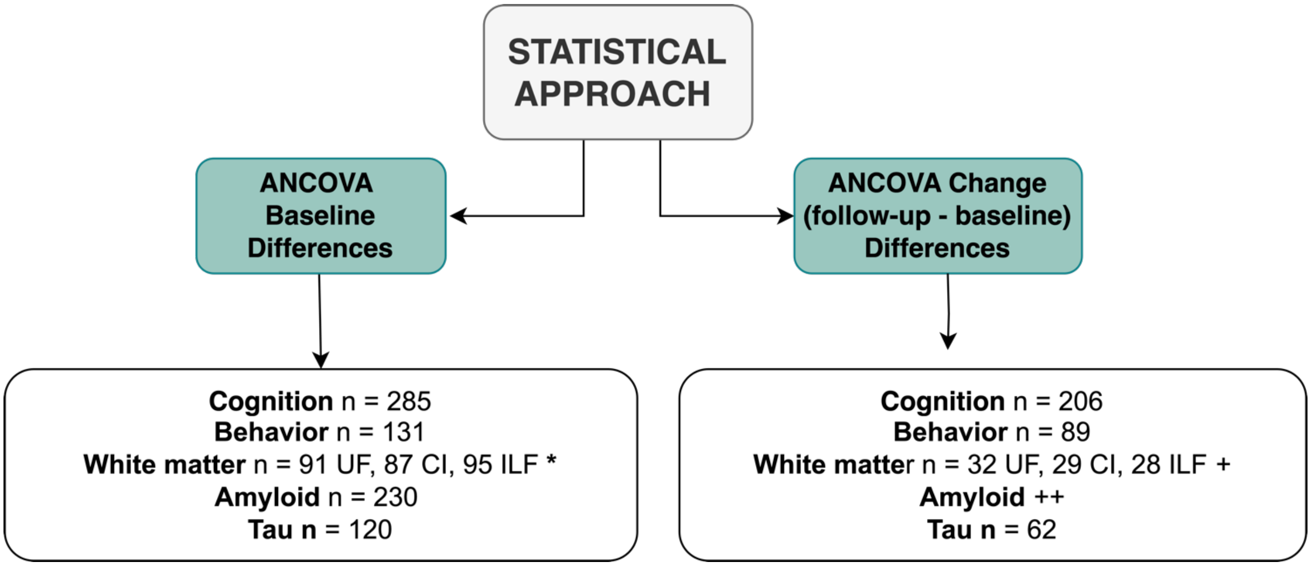

PTSD, posttraumatic stress disorder; TBI, traumatic brain injury; UF, uncinate fasciculus; CI, cingulum; ILF, inferior longitudinal fasciculus. For non-normally distributed variables, Kruskall-Wallis tests were run.

*TBI Group Missing (*n*= 8); + TBI and PTSD+TBI Groups Missing (*n* = 4 and *n* = 4); ++ Amyloid Missing (*n* = 9).

Supplementary Table 1. Variables, Statistical Tests and Hypotheses.

Domain	Outcome	Measurement	Statistical Test	Groups	Hypotheses
**BASELINE**					
Cognition	Boston Naming Test (reduced version - 30 items; BNT)<br>*Confrontation Naming*	Number of objects correctly named without cue + number of objects correctly named after cue (higher scores represent better confrontation naming abilities – 30 is the maximum)	ANCOVA	PTSD versus TBI versus PTSD+TBI versus Veteran controls	Lower scores expected in Veterans with PTSD, TBI, PTSD+TBI compared to Veteran controls.
	Trails Making Test B – A (derived score; TMT B-A)<br>*Processing speed and executive functioning*	Time, in seconds (lower score represents better performance)	ANCOVA	PTSD versus TBI versus PTSD+TBI versus Veteran controls	Higher scores expected in Veterans with PTSD, TBI, PTSD+TBI compared to Veteran controls.
	Rey Auditory Verbal Learning Test<br>(30 minutes delay; AVDEL)<br>*Episodic memory delayed*	Number of words remembered after a 30-min delay (higher score represents better delayed memory)	ANCOVA	PTSD versus TBI versus PTSD+TBI versus Veteran controls	Lower scores expected in Veterans with PTSD, TBI, PTSD+TBI compared to Veteran controls.
Behavior	Neuropsychiatric Inventory (NPI)<br>*Neuropsychiatric disturbances*	Sum of all domain scores, but does not include the distress score (higher score represents higher psychopathology)	Kruskall-Wallis	PTSD versus TBI versus PTSD+TBI versus Veteran controls	Higher scores expected in Veterans with PTSD, TBI, PTSD+TBI compared to Veteran controls.
	Functional Assessment Questionnaire (FAQ)<br>*Instrumental Activities of Daily Living (IADL)*	Sum of ratings given for each domain (higher score represents lower functional abilities)			Higher scores expected in Veterans with PTSD, TBI, PTSD+TBI compared to Veteran controls.
White matter	Uncinate Fasciculus (UF)	Fractional anisotropy	ANCOVA	PTSD versus PTSD+TBI versus Veteran controls	Lower scores expected in Veterans with PTSD and PTSD+TBI compared to Veteran controls.
	Cingulate (CI)	Fractional anisotropy	ANCOVA		See UF
	Inferior Longitudinal Fasciculus (ILF)	Fractional anisotropy	ANCOVA		See UF
Amyloid PET	Composite florbetapir cortical SUVR (weighted florbetapir mean in frontal, cingulate, parietal, and temporal regions, defined by Freesurfer)	SUVR	ANCOVA	PTSD versus TBI versus PTSD+TBI versus Veteran controls	Higher SUVR expected in Veterans with PTSD, TBI, PTSD+TBI compared to Veteran controls.

Tau PET	Temporal flortaucipir SUVR	SUVR	ANCOVA	PTSD versus TBI versus PTSD+TBI versus Veteran controls	Higher SUVR expected in Veterans with PTSD, TBI, PTSD+TBI compared to Veteran controls.
**FOLLOW-UP - BASELINE**					
Cognition	BNT, TMT B-A, AVDEL30	Follow-up - baseline	ANCOVA	PTSD versus TBI versus PTSD+TBI versus Veteran controls	Negative score difference expected in Veterans with PTSD, TBI, PTSD+TBI compared to controls, while positive for TMT B-A.
Behavior	NPI, FAQ	Follow-up - baseline	ANCOVA	PTSD versus TBI versus PTSD+TBI versus Veteran controls	Positive score difference expected in Veterans with PTSD, TBI, PTSD+TBI compared to controls.
White matter	UF, CI, and ILF	Follow-up - baseline	ANCOVA	PTSD versus Veteran controls	Negative score difference expected in Veteran with PTSD compared to controls.
Tau PET	Temporal flortaucipir SUVR	Follow-up - baseline	ANCOVA	PTSD versus TBI versus PTSD+TBI versus Veteran controls	Positive score difference expected in Veterans with PTSD, TBI, PTSD+TBI compared to controls.

ANCOVA, analysis of covariance; AVDEL30, Auditory Verbal Learning Test 30-Minutes Delayed; BNT, Boston Naming Test; CI FA, cingulate fractional anisotropy; FAQ, Functional Assessment Questionnaire; ILF FA, inferior longitudinal fasciculus fractional anisotropy; NPI, Neuropsychiatric Inventory; PTSD, posttraumatic stress disorder; SD, standard deviation; SUVR, standardized uptake value ratio; TBI, traumatic brain injury; TMT B-A, Trail Making Test B-A; UF FA, uncinate fasciculus fractional anisotropy.

Additional information on subjects with and subjects without available or analyzed DTI data

Supplementary Table 2. Baseline characteristics of subjects with and subjects without available or analyzed DTI data.

		With DTI (n=95)	Without DTI (n=190)	*p*
		**Mean ± SD**	**Mean ± SD**	***p***
**Age (y)**		69.58 ± 4.42	69.92 ± 4.40	0.540
**Education (y)**		15.35 ± 2.54	15.12 ± 2.36	0.443
**Life CAPS**		36.70 ± 33.83	46.33 ± 32.58	**0.021***
**BNT**		28.14 ± 2.07	27.69 ± 3.06	0.177
**TMT B-A**		61.64 ± 38.45	61.10 ± 43.02	0.917
**AVDEL30**		6.70 ± 3.50	5.38 ± 3.79	**0.005***
		%	***%***	***p***
**Cohort**	**PTSD**	29.3%	26.9%	**0.012***
	**TBI**	11.1%	15.4%	
	**PTSD+TBI**	25.3%	39.0%	
	**Control**	34.3%	18.7%	
**Genotype**	***APOE* ε4+**	29.8%	24.9%	0.384
**Ethnicity**	**Hispanic**	9.1%	7.7%	0.918
	**Caucasian**	89.9%	91.2%	
	**Unknown**	1%	1.1%	
**Race**	**American Indian**	1.0%	1.6%	0.803
	**Asian**	2%	0.5%	
	**African American**	9.1%	6.6%	
	**White**	82.8%	84.6%	
	**More than one race**	4%	5.5%	
	**Unknown**	1%	1.1%	
**Language**	**English**	99.0%	97.3%	0.524
	**Spanish**	1%	1.6%	
	**Other**	0%	1.1%	
**Work Status**	**Working**	17.2%	11.5%	0.187
	**Retired**	82.8%	88.5%	
**Handedness**	**Right**	88.5%	86.0%	0.929
	**Left**	11.5%	14.0%	
**Marital Status**	**Married**	83.8%	76.9%	0.444
	**Widowed**	4%	5.5%	
	**Divorced**	9.1%	10.4%	
	**Never married**	3%	7.1%	
**Medication usage**	**No psychiatric med**	43.5%	85.7%	0.578
	**Donepezil**	0%	2.4%	
	**Galantamine**	1.4%	2.4%	
	**Anti-depressants**	44.9%	7.1%	
	**Other psychiatric med**	10.1%	2.4%	
**Diagnosis**	**Mild cognitive impairment**	4.6%	25%	**<0.001***

Results are based on the data available for the ILF. AVDEL30, Auditory Verbal Learning Test 30 Minutes Delayed; BNT, Boston Naming Test; CAPS, Clinician Administered PTSD Scale.

Additional analyses PET data

The PET data available on the ADNI database is already intensity normalized, although Jagust's laboratory recommends intensity normalizing the SUVRs results in their dataset using a reference region in their dataset as explained here UCBERKELEY_AV1451_Methods_2021-01-14.pdf (adni.bitbucket.io).

For Florbetapir data we used two variables already available in the "UCBERKELEYAV45_20190808" file, namely "SUMMARYSUVR_WHOLECEREBNORM" (summary florbetapir cortical SUVR normalized by whole cerebellum) and "SUMMARYSUVR_COMPOSITE_REFNORM" (summary florbetapir cortical SUVR normalized by composite reference region).

For Flortaucipir data we divided the "METATEMPORAL_SUVR" by the "INFCEREB_SUVR" (reference region – av1451 mean of inferior cerebellar grey matter). No differences were shown in any of the PET analyses using the abovementioned variables among the four groups (p>0.05, Supplementary Table 3).

Supplementary Table 3. SUVR relative to reference region.

Analysis<br>Dependent variables		PTSD		TBI		PTSD+TBI		Control		TOTAL	*p*
**Kruskal Wallis+**	**n**	**Mean Rank**	**n**	**Mean Rank**	**n**	**Mean Rank**	**n**	**Mean Rank**	**n**		***p***
**Florbetapir composite**[1]	70	102.73	32	107.64	61	126.87	67	122.25	230		0.14
**ANCOVA**	**n**	**Mean ± SD**	**n**	**Mean ± SD**	**n**	**Mean ± SD**	**n**	**Mean ± SD**	**n**	**Mean ± SD**	***p***
**Florbetapir composite**[2]	70	0.76±0.07	32	0.78±0.10	61	0.79±0.10	67	0.79±0.10	230	0.78±0.09	0.44
**Flortaucipir temporal**[3]	38	1.16±0.08	18	1.18±0.08	37	1.16±0.10	27	1.17±0.07	120	1.17±0.09	0.84
**Flortaucipir temporal change**[4]	19	-0.01±0.04	10	-0.02±0.03	20	0.01±0.05	13	-0.00±0.03	62	0.01±0.04	0.24

[1]SUVR, normalized by whole cerebellum; [2]SUVR, normalized by composite reference region; [3]SUVR, normalized dividing the temporal summary region by reference region; [4]SUVR, normalized temporal region change (follow-up – baseline); +due to skewness= 2.13; PTSD, posttraumatic stress disorder; SD, standard deviation; TBI, traumatic brain injury. Data corrected for age and education.

Supplementary Figures 2 and 3. Scatterplots displaying the association between difference scores and time interval between assessments.

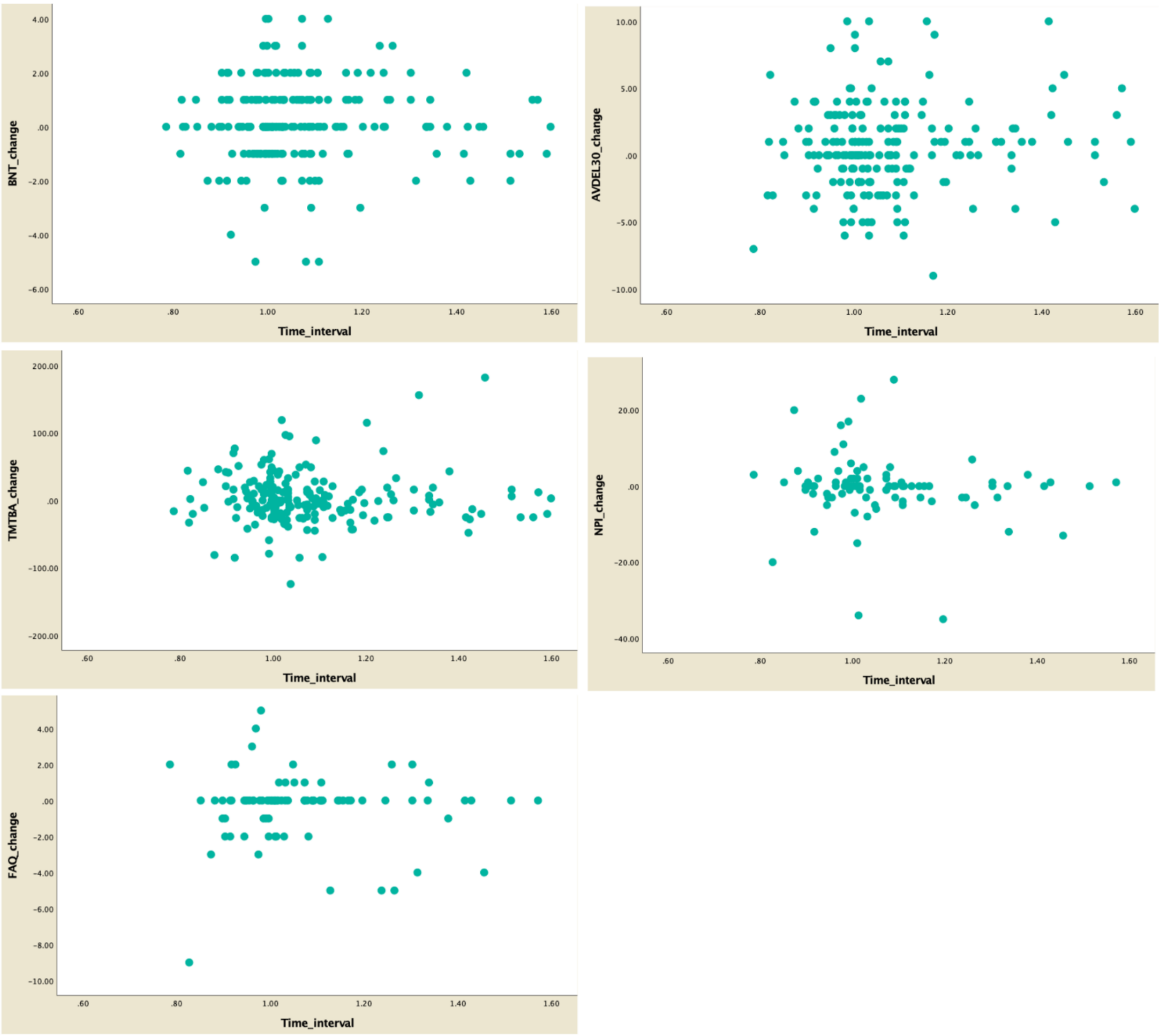

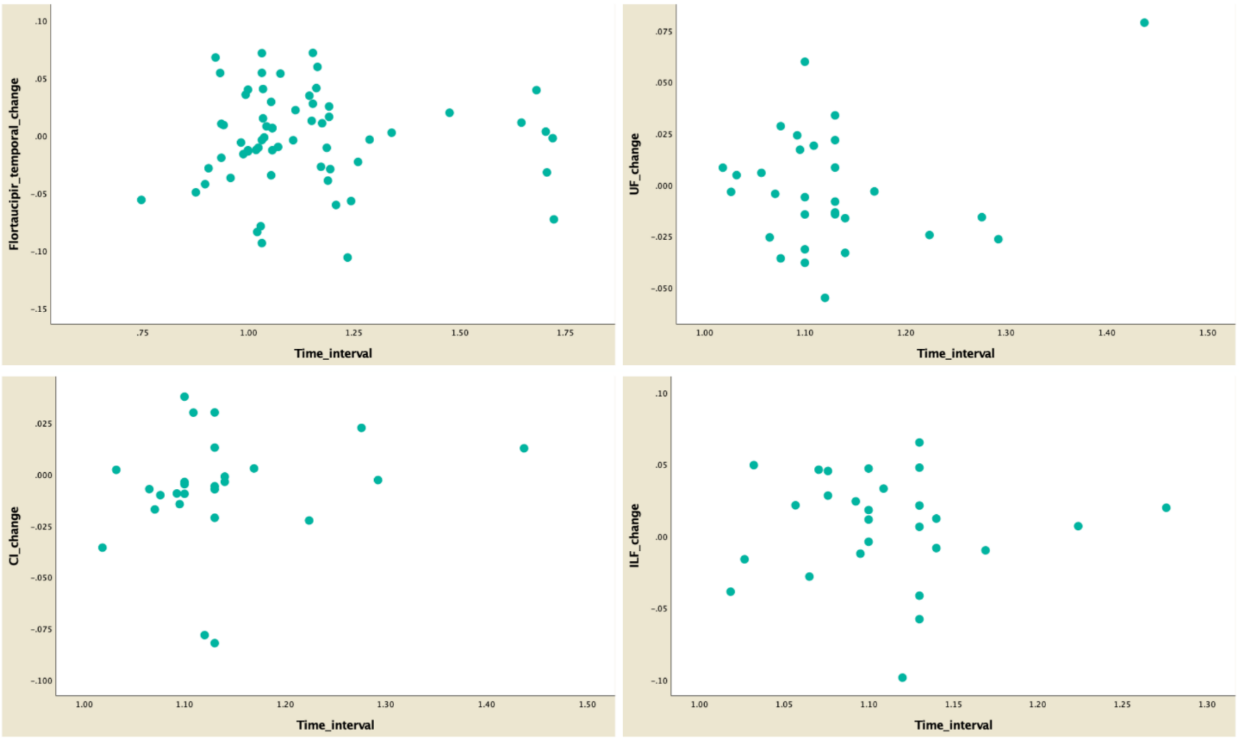

The bivariate Pearson correlations assessing the association between difference scores and time interval were non-significant ($p > 0.05$) for all nine outcome measures.
